# Clinical next-generation sequencing reveals aggressive cancer biology in adolescent and young adult patients

**DOI:** 10.18632/oncoscience.176

**Published:** 2015-07-08

**Authors:** Vivek Subbiah, Manojkumar Bupathi, Shumei Kato, Andrew Livingston, John Slopis, Pete M. Anderson, David S. Hong

**Affiliations:** ^1^ Department of Phase I Investigational Cancer Therapeutics, The University of Texas MD Anderson Cancer Center, Houston, Texas, USA; ^2^ Division of Cancer Medicine, The University of Texas MD Anderson Cancer Center, Houston, Texas, USA; ^3^ Department of Neuro-oncology, Division of Cancer Medicine, The University of Texas MD Anderson Cancer Center, Houston, Texas, USA; ^4^ Pediatric Hematology Oncology and Blood and Marrow Transplantation, Cleveland Clinic Foundation, Cleveland, OH, USA

**Keywords:** next generation sequencing, adolescents and young adults, cancer biology, AYA, mTOR, AKT, TP53

## Abstract

**Background:**

The aggressive biology of cancers arising in adolescent and young adult (AYA; ages 15–39 years) patients is thought to contribute to poor survival outcomes.

**Methods:**

We used clinical next-generation sequencing (NGS) results to examine the molecular alterations and diverse biology of cancer in AYA patients referred to the Phase 1 program at UT MD Anderson Cancer Center.

**Results:**

Among the 28 patients analyzed (14 female and 14 male), 12 had pediatric-type cancers, six had adult-type cancers, and ten had orphan cancers. Unique, hitherto unreported aberrations were identified in all types of cancers. Aberrations in *TP53*, *NKX2-1*, *KRAS*, *CDKN2A*, *MDM4*, *MCL1*, *MYC*, *BCL2L2*, and *RB1* were demonstrated across all tumor types. Five patients harbored *TP53* aberrations; three patients harbored *MYC*, *MCL1*, and *CDKN2A* aberrations; and two patients harbored *NKX2-1*, *KRAS*, *MDM4*, *BCL2L2*, and *RB1* alterations. Several patients had multiple aberrations; a patient with wild-type gastrointestinal stromal tumor harbored five alterations (*MDM4*, *MCL1*, *KIT*, *AKT3*, and *PDGRFA*).

**Conclusions:**

This preliminary report of NGS of cancer in AYA patients reveals diverse and unique aberrations. Further molecular profiling and a deeper understanding of the biology of these unique aberrations are warranted and may lead to targeted therapeutic interventions.

## INTRODUCTION

Over the past few decades, treatment for aggressive cancers in both adult and pediatric patients has substantially improved, thus increasing survival rates for these groups [1]. Unfortunately, improvements in cancer treatment for adolescent and young adult (AYA) patients, specifically those between the ages of 15 and 39 years (the age generally used in the United States), have lagged behind [2]. Cancer remains a leading cause of death in this population, behind only homicide, suicide, and injuries [3,4]. The most common malignancies in this age group are lymphoma, melanoma, testicular cancer, thyroid cancer, sarcoma, leukemia, central nervous system tumors, and breast cancer [2].

Recently, increasing awareness has led to interest in defining the psychosocial and host-related factors that contribute to the discrepant and poor outcomes observed in AYA patients. Multiple authors have examined the effects of psychosocial factors, poor medication compliance, and under-insurance on outcomes [2,5]. These psychosocial causes are compounded by host-related factors such as decreased tolerance to intensive chemotherapy regimens, comorbidities, and unique pharmacodynamics that accompany puberty and are a part of the normal aging process. Clinical trial enrollment poses additional challenges within this demographic group; on average about 10% of patients between the ages of 15 and 19 years-old and only about 1–2% of patients between the ages 20 and 39 years-old are enrolled in a clinical trial [2].

However, even with the increased awareness of poor prognosis among AYA patients with cancer, the underlying biology of the diseases at a molecular and genomic level remains largely unstudied [6] The aggressive biology of cancer in AYAs is cited as one of the reasons for poor prognosis in this age group [5, 7]. In addition, because AYA patients face challenges related to decreased access to healthcare and lack of insurance, they are less likely to be seen in research institutions, contributing to lower rates of clinical trial enrollment among AYA patients [8]. Moreover, few clinical trials target this age group. As a result, there are fewer tumor tissue banked specimens available for research from AYA patients than from other age groups, leading to little knowledge of host biology in the AYA population [9].

Thus, studies of the biology of cancers in AYAs are urgently needed. Understanding the biology could lead to future clinical trials of agents targeting the aberrant pathways of cancers in AYAs. In this study, we report a preliminary analysis of next-generation sequencing (NGS) profiles of all AYA patients referred to the Center for Targeted Therapy, a phase I clinical trials program at our institution. This report aims to improve understanding of tumor biology and identify possible actionable mutations. We report 17 different tumor types in 28 patients with diverse and heterogeneous malignancies characterized by aggressiveness.

## RESULTS

A total of 28 AYA patients were seen during the period studied (Table 1). The median age was 23 years-old (range: 18–39); 14 patients (50%) were female and 14 (50%) were male. The median number of prior therapies for metastatic disease was three (range: 1–11).

**Table 1:** Characteristics of patients included in the analysis (*N*=28)

Characteristic	Number of patients (%)
Gender	
Male	14 (50)
Female	14 (50)
ECOG performance status	
<1	10 (36)
≥1	18 (64)
Number of prior therapies	
≤3	15 (54)
>3	13 (46)
Diagnosis	
Osteosarcoma	5 (17)
Medulloblastoma	2 (7)
Alveolar rhabdomyosarcoma	3 (11)
Neuroblastoma	1 (4)
Wilms tumor	1 (4)
Non-small cell lung cancer	1 (4)
Colorectal cancer	1 (4)
Nasopharyngeal carcinoma	2 (7)
Triple-negative breast cancer	1 (4)
Gastric adenocarcinoma	1 (4)
Renal medullary carcinoma	2 (7)
Fibrolamellar hepatocellular carcinoma	2 (7)
Juvenile hyaline fibromatosis	1 (4)
Malignant peripheral nerve sheath tumor	1 (4)
Neurofibromatosis type II	1 (4)
Small cell sarcoma	1 (4)
Gastrointestinal stromal tumor	1 (4)
Neuroendocrine carcinoma of lung	1 (4)

ECOG, Eastern Cooperative Oncology Group

Among the 28 patients, 17 different tumor types were reported, with sarcoma the most common malignancy. Mutations were identified in 21 patients; 16 of these patients had more than one mutation. The most common molecular aberrations were *TP53*, *NKX2-1*, *KRAS*, *CDKN2A*, *MDM4*, *MCL1*, *MYC*, *BCL2L2*, and *RB1* (Table 2). These abnormalities were observed in non-small lung cancer, colon cancer, osteosarcoma, breast cancer, small cell sarcoma, alveolar rhabdomyosarcoma, neuroendocrine carcinoma of the lung, neurofibromatosis II, gastrointestinal stromal tumor (GIST), and nasopharyngeal carcinoma. Five patients (19%) had a *TP53* mutation, three (11%) had *MYC* mutations, and two (7%) had a *FBXW7* mutation (Table 3).

**Table 2:** Clinical and molecular characteristics in AYA patients and recommended therapies

Tumor type	Aberrations identified	Tested site	Recommended therapy	Matched therapy	Response
Juvenile hyaline fibromatosis	none	bone	vandatenib and everolimus	no	n/a
Metastatic medulloblastoma	*BRCA*	liver	HAI irinotecan, bevacizumab, and cetuximab	no	n/a
Metastatic osteosarcoma	*TP53, NKX2-1, MYC, BCL2L2, RB1*	lung	bevacizumab, temsirolimus, and valproic acid	yes	no
Metastatic non-small cell lung cancer	*EGFR, TP53, NKX2-1, KRAS*	lung	docetaxel and erlotinib	yes	no
Diffuse type gastric adenocarcinoma	increased c-Met	stomach	FOLFIRI with cetuximab	no	n/a
Non-small cell lung cancer	*EGFR, TP53, NKX2-1* amplification	lung	docetaxel and erlotinib	yes	no
Fibrolamellar hepatocellular carcinoma	none	diaphragm	sunitinib and valproic acid	no	n/a
Fibrolamellar hepatocellular carcinoma	*FBXW7*	liver	sirolimus and vorinostat	no	n/a
Renal medullary carcinoma	none	kidney	pazopanib and crizotinib	no	n/a
Wilms tumor	*CTNNB1, IGF1R, FAM123B, SPEN*	kidney	vincristine, actinomycin, and doxorubicin	no	n/a
Medullary renal cell carcinoma	none	kidney	gemcitabine and adriamycin	no	n/a
Neuroendocrine carcinoma of lung	*CCND1, MYCL1, MDM2, MLL2, FGF19/3/4*	mediastinum	vandetanib and everolimus	no	n/a
Nasopharyngeal carcinoma	*AKT2, PIK3R2, PALB2*	lymph node	poor PS	no	n/a
Alveolar rhabdomyosarcoma	none	lymph node	vincristine and irinotecan	no	n/a
Nasopharyngeal carcinoma	*MCL1*	nasopharynx	sirolimus and cetuximab	no	n/a
Alveolar rhabdomyosarcoma	*CDKN2A, MCL1* amplification, *PAX3, FKHR*	lung	temsirolimus and metformin	no	n/a
Metastatic osteosarcoma	*MYC, DMT3A*	bone	pazopanib + pemetrexed, then pazopanib + crizotinib	no	n/a
Neurofibromatosis II	*MCL1*	brain	bevacizumab and temsirolimus	no	yes
Metastatic colon cancer	*TP53, KRAS, CCNE1, CRKL*	colon	FOLFIRI with cetuximab	yes	no
Ewing sarcoma	*EWSR1-FLI1*	lymph node	PARP inhibitor (BMN673)	yes	no
Neuroblastoma/paraganglioma	Overexpression of p-AKT and p-ERK*	adrenal mass	vandatenib and everolimus	yes	no
Metastatic osteosarcoma	*HER2/neu*	bone	metformin and lapatinib	yes	no
Metastatic osteosarcoma	*TP53, PIK3CA, RB1, JUN Amp, HER2/neu*	lung	pazopanib plus lapatinib	yes	no
Alveolar rhabdomyosarcoma	none	bone	pazopanib and crizotinib	no	n/a
Metastatic medulloblastoma	PTCH1, negative for SHH	spine	ICE protocol with intrathecal topotecan/liposomal cytarabine	no	n/a
Triple negative breast cancer	*TP53, MYC, FBXW7*	breast	MK-886 (mTOR inhibitor) MK-2206 (AKT inhibitor)	yes	yes
Metastatic gastrointestinal stromal tumor	*MDM2, MCL1, KIT, AKT3, PDGFRA*	abdomen	sunitinib	yes	no

FOLFIRI, Folinic acid, fluorouracil and irinotecan; HAI, Hepatic artery infusion; ICE, Ifosfamide, carbop; n/a, not applicable; PS, performance status.

* By immunohistochemistry.

**Table 3:** Common Abnormalities

Abnormality	Number of patients (%)
*TP53*	5 (17)
*MYC*	3 (11)
*MCL1*	3 (11)
*CDKN2A*	3 (11)
*NKX2-1*	2 (7)
*KRAS*	2 (7)
*MDM4*	2 (7)
*BCL2L2*	2 (7)
*RB1*	2 (7)
*FBXW7*	2 (7)

At the time of analysis, 15 patients were still alive and receiving treatment. Nine were placed on a molecularly matched therapy based on their FoundationOne analysis results, and this matched therapy was either directly or indirectly targeted (see Table 2 for further details). Remaining six patients did not receive matched therapy due to their comorbidities or lack of available clinical trials for matched therapies.

## DISCUSSION

Cancers affecting AYA patients are diverse, spanning the spectrum from pediatric to adult-type malignancies. Young women aged 15–39 years-old are more likely to have high-grade, locally advanced triple-negative breast cancer, [10] and young age appears to be a specific indicator of poor prognosis for this disease, independent of stage or histologic type [11]. Thus identifying and characterizing the genomic aberrations among cancers in AYAs may help understand the role of disease biology in determining prognosis and predicting therapeutic outcomes. We retrospectively reviewed the NGS results of pediatric- and adult-type cancers, as well as types that are considered cancers primarily experienced by AYAs.

In our analysis, one patient had triple-negative breast cancer with prognostic and potentially targetable alterations in *TP53*, *MYC*, and *FBXW7*. This shows that young patients with triple-negative breast cancer should be screened with molecular testing and that targeted agents are a potential treatment option.

In patients with Wilms tumor—a pediatric malignancy more common in young children than in adolescents or young adults—survival outcomes for those with early-stage disease are similar between pediatric and AYA patients, but for those with advanced-stage disease, worse outcomes have been reported among AYA patients than in pediatric patients [12]. As with other malignancies, the role of disease biology in these observations remains unclear. Our NGS analysis of a Wilms tumor sample from one AYA patient with advanced disease revealed multiple aberrations, including *CTNNB1*, *IGF1R*, *FAM123B*, and *SPEN*. Recently, it was shown that increased DNA copy number and expression of *IGF1R* are associated with poor outcome in patients with Wilms tumors [13]. and that these mutations may be targetable by specific receptor inhibition [14]. It is unknown if AYA patients with Wilms tumors are more likely to have IGF1R mutations than other patient populations or if AYA patients may benefit from IGF1-targeted therapies.

In our cohort, we had two patients with fibrolamellar hepatocellular carcinoma (FL-HCC), a rare tumor that is poorly understood and most commonly occurs in AYA patients [15,16]. Honeyman et al. recently described the presence of *DNAJB1-PRKACA* chimeric transcript in 100% of all patients who were tested. This chimeric protein was not present in any normal liver cells that were tested. It is currently understood that this chimeric transcript contributes to the pathogenesis of FL-HCC, although the exact role is unknown [17]. Our patients were not tested for this particular chimeric protein. Furthermore, although our first patient's molecular testing did not reveal any aberrations, the second patient had a *FBXW7-E192A* alteration and was thus treated with sirolimus and vorinostat. The latter patient had stable disease for a period of 6 months. Although our experience indicate *FBXW7-E192A* alteration may be associated with disease stabilization using sirolimus and vorinostat, Jardim et al. showed somatic mutations in FBXW7 usually occurred with other molecular aberrations, thus limiting the efficacy of mTOR inhibitors [18]. Further investigation is warranted.

KIT and platelet-derived growth factor receptor-β mutations are demonstrated in the overwhelming majority of adults with GIST, and approximately 80% of adult patients have KIT gene mutations that leads to constitutive activation of the KIT receptor [19]. These aberrations have been targeted with tyrosine kinase inhibitors such as imatinib. In contrast, young patients with GIST are far less likely to exhibit these mutations and have therefore shown little benefit from tyrosine kinase inhibitors [20]. Recently, so-called pediatric-type GIST has been described in adults, arising as multifocal tumors that frequently metastasize to the lymph nodes and generally follow an indolent course despite imatinib resistance [21]. These observations from mutational analyses serve as a basis for considering alternative treatments for GIST in the AYA population. In our study, one patient had wild-type GIST harboring multiple aberrations, including *KIT*, as well as *MDM4*, *MCL1*, *AKT3*, *and PDGFRA*.

We also observed commonly described mutations in our AYA patient cohort. Mutations in the *TP53* tumor suppressor gene are the most common genetic mutations identified in human cancers, and this was true in our analysis as well. Rates of somatic *TP53* mutations in sporadic cancers range from 10% to 60% [22]. We observed *TP53* mutations in 19% of the patients in our study. It has been shown that *TP53* mutations have prognostic significance in certain types of AYA tumors and may help guide therapeutic strategies. For example, one study of early-onset breast cancer that included AYA patients, *TP53* mutations were significantly more common among women with a strong family history of breast cancer [23]. *TP53* mutations have been further shown to have a strong association with basal-like and HER2+ breast cancer, both of which carry a poorer prognosis than other types of breast cancer [24]. In contrast, the lack of *TP53* mutations in testicular germ cell tumors is thought to partially account for their chemosensitivity to platinum-based drugs through *TP53*-mediated apoptosis [25].

All patients whose records were included in our descriptive analysis were referred for evaluation and treatment on a phase I clinical trial at a major cancer center and had experienced progression of metastatic disease while receiving at least one line of systemic therapy. Only records from patients who had received complete FoundationOne NGS analysis were included in the study. These limitations pose a referral bias and a potential selection bias toward aggressive cancers. Although our analysis included bone and soft tissue sarcomas, breast cancer, and central nervous system tumors, the analysis did not include many of the most common cancers affecting the AYA population, notably hematologic malignancies, melanoma, thyroid cancer, and testicular germ cell tumors.

Although most patients whose records were examined in our study had potentially targetable aberrations identified by FoundationOne analysis, only nine patients were treated with matched therapies. Our institution has previously reported that patients treated in phase I clinical trials with matched therapies have better outcomes, including higher response rates, longer times to treatment failure, and longer survival, compared to those not treated with matched therapies [26]. The limited number of clinical trials of targeted therapies available to AYA patients poses a further challenge to treating AYA patients with matched therapies even when targetable mutations are identified. One of the major limitations of this study is that the sequencing involved a panel based approach as opposed to a whole exome or whole genome approach. But this is a retrospective review of patients presenting with clinical next generation sequencing and perhaps it would be worthwhile to prospectively study these patients with whole exome or a whole genome approach.

Although no clinical trials are currently enrolling only AYA patients, recent efforts have focused on designing clinical trials with inclusion criteria that span pediatric and adult patients and are not limited by age. Within our institution, specific efforts are underway to develop a phase I trial program within the Department of Investigational Cancer Therapeutics, in collaboration with the Department of Pediatrics, for AYA patients. Currently, a phase I dose escalation trial of radium 223 for osteosarcoma (NCT01833520) and a phase I dose escalation trial of vandetanib and everolimus (NCT01582191) that allows enrollment of AYA patients are underway. We hope to open additional phase I trials to address the unique needs of the AYA population. Furthermore, we are currently performing CLIA-certified molecular testing on all newly referred patients to identify targetable molecular aberrations for matched therapies.

## CONCLUSION

AYA patients usually have aggressive tumors and ongoing studies are needed to better define the distinctive disease biology of these tumors and to delineate the degree to which age-specific disease biology may contribute to adverse outcomes. Ideally, analysis of the genomics of cancers in AYAs could be compared to analysis of tumors of the same cancer types in pediatric and adult patients to identify clinically relevant differences in disease biology. As molecular profiling and NGS technology such as with whole genome and/or exome sequencing become more readily available, investigation may reveal unique driver mutations with therapeutic implications, guiding clinicians in the use of pediatric- or adult-like treatment regimens or possibly novel targeted therapeutics for cancer in AYA patients.

## METHODS

We conducted a retrospective analysis of medical records in the Center for Targeted Therapy's phase I clinical trials program at the University of Texas' MD Anderson Cancer Center using our electronic medical system, ClinicStation. Records included in this study were from patients seen in our department between June 2012 and May 2013 who were in the AYA age group of 15–39 years-old, had experienced progression of metastatic disease while receiving at least one line of systemic therapy, and had undergone FoundationOne genomic analysis. We collected various demographic and clinicopathologic data and the FoundationOne analysis results for each patient. We did not perform any formal statistical hypothesis testing for this study; descriptive statistics were used to summarize the data.

FoundationOne is a commercially-available, Clinical Laboratory Improvement Amendments (CLIA)-certified targeted sequencing assay that uses NGS technology. NGS is a diagnostic tool used to detect somatic mutations in cancer cells to aid in providing personalized treatment. Frampton et al. published a validation study for the use of NGS based on parallel DNA sequencing by using 53 cell lines to create three types of reference materials designed to identify base substitutions, indels (short insertions or deletions), or copy number variations. The validation sensitivity achieved was 95–99% across all alteration types, with a positive predictive value >99% [27]. FoundationOne at the time when this analysis was conducted included 236 cancer-related genes and uses the mechanism that was validated by Frampton et al. to identify actionable genomic alterations in multiple solid tumor types [27].
